# Long Non-coding RNA *LINC02195* as a Regulator of MHC I Molecules and Favorable Prognostic Marker for Head and Neck Squamous Cell Carcinoma

**DOI:** 10.3389/fonc.2020.00615

**Published:** 2020-05-06

**Authors:** Hao Li, Hong-Gang Xiong, Yao Xiao, Qi-Chao Yang, Shao-Chen Yang, Hong-Chao Tang, Wen-Feng Zhang, Zhi-Jun Sun

**Affiliations:** ^1^The State Key Laboratory Breeding Base of Basic Science of Stomatology (Hubei-MOST) & Key Laboratory of Oral Biomedicine Ministry of Education, School & Hospital of Stomatology, Wuhan University, Wuhan, China; ^2^Department of Oral and Maxillofacial Head Neck Surgery, School & Hospital of Stomatology, Wuhan University, Wuhan, China

**Keywords:** lncRNA, MHC, TCGA, tumor microenvironment, T cell

## Abstract

The loss of major histocompatibility complex class I (MHC I) molecules is an important mechanism by which cancer cells escape immunosurveillance in head and neck squamous cell carcinoma (HNSCC). Several long non-coding RNAs (lncRNAs) have been implicated in immune response and regulation including antigen processing and presentation. However, few studies on lncRNAs regulating MHC I expression in HNSCC have been conducted. In this study, MHC I related lncRNAs were identified from the The Cancer Genome Atlas (TCGA) HNSCC database. One of the lncRNAs, *long intergenic non-protein coding RNA 2195* (*LINC02195*), was found to be associated with genes encoding MHC I molecules and patient prognosis in the TCGA database. KEGG and GO analyses suggested that *LINC02195* was closely related to antigen processing and presentation. qRT-PCR revealed high expression of *LINC02195* in human HNSCC tissues and HNSCC cell lines compared with normal mucosal tissues. *in situ* hybridization of the HNSCC tissue microarray revealed a correlation between high *LINC02195* expression and a favorable prognosis in our patient cohort. Silencing of *LINC02195* decreased MHC I protein expression, as evidenced by western blotting. Multiplex immunochemistry was performed to reveal the positive correlation between high *LINC02195* expression and an increased number of CD8^+^ and CD4^+^ T cells in the tumor microenvironment. Based on our study, *LINC02195* is a promising prognostic marker and a target for future therapeutic interventions.

## Introduction

Head and neck squamous cell carcinoma (HNSCC) accounts for 90% of all cases of head and neck cancers, which causes ~430,000 deaths worldwide (1). HNSCC normally occurs in epithelial cells, including the mucosal lining of the upper airway and food passages (2). The general primary treatments for HNSCC include surgery, radiotherapy, chemotherapy and targeted therapy (3). In addition to these traditional treatments, immune checkpoint blockades such as anti-programmed cell death 1 (anti-PD-1) and anti-programmed cell death 1 ligand 1 (anti-PD-L1) antibodies have displayed great efficacy in the treatment of HNSCC (3, 4). However, the response rate to immune checkpoint blockades remains poor (3, 5). To increase the response rate, we should reveal the immunosuppressive mechanisms of tumors.

One of the roles of the innate and adaptive immune systems is to eliminate tumors before they are detectable. One of the mechanisms by which malignant cells escape immunosurveillance and elimination is the loss or downregulation of major histocompatibility complex class I (MHC I) molecules, limiting the recognition of tumor antigens by cytotoxic CD8^+^ T cells, which attack malignant cells (6). MHC I molecules are encoded by *human leukocyte antigen class I* (*HLA I*) genes mainly including *HLA-A, HLA-B*, and *HLA-C*, and are expressed by all somatic cells in humans (7). The deregulation of MHC I has been observed in various forms of cancer (8–10). Patients with lower levels of MHC I expression have poor survival for HNSCC (10). In addition, downregulation of MHC I in cancer cells was recently shown to be a mechanism underlying the immunotherapy resistance of cancer (11).

Long non-coding RNAs (lncRNAs) comprise various RNAs that are <200 nucleotides in length and have no protein-coding capacity. Several lncRNAs are abnormally expressed in various forms of cancer and are involved in the occurrence and development of tumors (12–14). With an increased understanding of lncRNAs, the important value of lncRNAs in treating tumors or predicting the prognosis of patients with tumors has been recognized (15). Notably, studies have recently been reported that some lncRNAs are identified as regulators of human immune system, particularly in anti-tumor immunity (16–18). A recent study discovered the association between lncRNAs and MHC I molecules. According to previous gene expression studies, the expression of the lncRNA *HCP5* is associated with *HLA-B* expression (19). The lncRNA *HOTAIR* was recently shown to promote *HLA-G* expression in gastric cancer (20). However, few studies on lncRNAs regulating MHC I expression in HNSCC have been performed.

Here, several differentially expressed lncRNAs were identified by analyzing of The Cancer Genome Atlas (TCGA) database. Furthermore, we investigated a highly expressed lncRNA, *long intergenic non-protein coding RNA 2195* (*LINC02195*, ENSG00000236481), which is closely associated with *HLA-A, -B, and -C* expression. Next, we investigated the biological function of *LINC02195* using bioinformatic analysis based on TCGA. A human tissue microarray (TMA) and *in situ* hybridization (ISH) were used to reveal the clinical role of *LINC02195*, and patients with high *LINC02195* expression achieved a good outcome in the HNSCC patient cohort. As shown in western blots, *LINC02195* silencing decreased the expression of MHC I molecules. By performing multiplex staining, a significant correlation between *LINC02195* and CD8^+^ and CD4^+^ T cell infiltration in the HNSCC microenvironment was revealed.

## Materials and Methods

Detailed information about the material and methods is provided in the Supplementary Material.

### Study Population, RNA Expression Data, and Bioinformatic Analysis

The RNA expression data for HNSCC cases, which included 502 HNSCC tumor samples and 44 normal tissue samples were acquired from the TCGA database derived from the data portal (https://gdc.cancer.gov/). The dataset included the expression of RNA (mRNA and non-coding RNA) (level 3) and clinical data from 546 individuals. RNAs were identified using the Ensembl database. The differentially expressed lncRNAs (DElncRNAs) and mRNAs (DEmRNAs) were identified using the “edgeR” package. DEIncRNAs and DEmRNAs were analyzed by constructing a volcano plot with the “ggplot2” package in the R language.

### Gene Enrichment and Functional Annotation Analysis

A subsequent functional enrichment analysis of the mRNAs (*q* values ≥ 0.4) was performed. The bubble map was drawn using the “ggplot2” R package. The mRNAs with significant Pearson's correlation coefficient values (|Pearson's correlation coefficient| ≥ 0.4) were included in further functional enrichment analyses. The Gene Ontology (GO) and Kyoto Encyclopedia of Genes and Genomes (KEGG) analyses were performed using the “clusterProfiler” package. The significant GO terms and KEGG pathways were identified as *LINC02195*-related biological functions and signaling pathways. Hierarchical clustering was performed using the “pheatmap” package in R language.

### Human HNSCC Samples and Analysis

Ethical approval for this study was obtained from the Medical Ethics Committee of the School and Stomatology of Wuhan University (PI: Zhi-Jun Sun; 2014LUNSHENZI06). Human HNSCC samples were obtained from the Department of Oral and Maxillofacial Surgery, School and Hospital of Stomatology Wuhan University. The TNM classification at diagnosis was determined according to the 8th edition of TNM Classification of Malignant Tumors (UICC). The patient cohort included 5 patients with normal oral mucosae, 28 with oral epithelial dysplasia and 59 with HNSCC and 10 paired fresh HNSCC samples. The clinical characteristics, including TNM classification, histological grade and overall survival were available for all patients.

ISH was performed on a human HNSCC TMA with digoxigenin-labeled antisense oligonucleotide probes to examine the expression of *LINC02195*, as previously described (21). The probe sequence for *LINC02195* was 5′-DIG-TCCTTTGGAATCCTCCTACTTTGGCAGC-3′. IHC staining was performed as described (22). Signals were detected using biotinylated goat anti-rabbit or anti-mouse antibody followed by streptavidin HRP. Staining was visualized with DAB (Dako, USA), counterstained with hematoxylin (Dako), sealed with neutral resins, and imaged. The scanning of the TMA and processing of histoscores were performed using previously described methods (22). A human leukocyte antigen (HLA) class I ABC antibody (1:300, Proteintech, USA) was used to detect MHC I molecules in human HNSCC samples.

### Cell Lines, siRNAs, and Western Blotting

The cell lines SCC4, SCC9, and CAL27 were obtained from ATCC (American Type Culture Collection) and maintained as previously described (23). TCA8113 cells were acquired from the Ninth People's Hospital, Shanghai Jiao Tong University and maintained in DMEM containing 1% penicillin and streptomycin (Thermo Fisher, USA) and 10% fetal bovine serum (FBS, Gibco, USA). The human oral keratinocyte cell line (HOK) was obtained from ScienCell.

Small interfering RNAs (siRNAs) targeting *LINC02195* were purchased from GenePharma (China). SCC9 cells seeded in a 6-well plate were transfected with the siRNAs using Lipofectamine 3,000 (Invitrogen, USA) according to the manufacturer's instructions.

Western blotting with whole-cell protein extracts from SCC9 cells was performed as previously described (21). An HLA class I ABC antibody (15240-1-AP; Proteintech, USA) was used for western blotting. GAPDH served as an internal loading control. All western blots were performed three times.

### Total RNA Extraction and Quantitative Reverse Transcription Polymerase Chain Reaction (qRT-PCR) Analysis

The total RNA extraction protocol and qRT-PCR analysis have been described previously (21). *LINC02195* expression was calculated with the comparative Ct method (2^−ΔΔCT^) and normalized to GAPDH expression. All qRT-PCR experiments were performed three times.

### Multiplex IHC and Image Analysis

The Opal 7-Color Manual IHC Kit (NEL811001KT; PerkinElmer, Hopkinton, MA, USA) was used to stain the TMA as previously described (24). Briefly, for CD4, CD8, PD-1, and pan-keratin 4-plex staining, 4 types of tyramide system amplification (TSA) sensors (Opal520, Opal570, Opal620, and Opal690) were used. After all multiplex TSA staining protocols was completed, the slide was stained with DAPI and mounted. All slides were scanned using the PerkinElmer Vectra system (PerkinElmer). The tumor and stromal area were distinguished and calculated with inForm (inForm 2.1.1; PerkinElmer) software using pan-keratin as a tumor marker. From these data, the positive cell density was calculated using the following formula: positive cell density = the number of positive cells in the stroma ÷ the area of the stroma (calculating by image pixels).

### Statistical Analysis

All data were statistically analyzed using Prism 8 software (GraphPad Software). One-way ANOVA (> 2 groups) or Student's *t*-tests (2 groups) (> 2 groups) were used to determine the significance of differences. Correlations were determined by calculating Pearson's correlation coefficient. Kaplan-Meier curves and Cox proportional hazards models were used to assess the prognostic value. Error bars are shown in the figures, and data are presented as the mean ± SEM. *P* < 0.05 was considered to be statistically significant, ^*^P < 0.05, ^**^P < 0.01, and ^***^P < 0.001.

## Results

### *LINC02195* Was Closely Correlated With *HLA I* Expression in the TCGA Database

**Figure 1:**
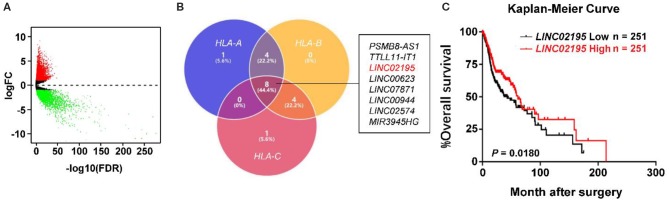
*LINC02195* expression was closely correlated with MHC I molecules in the TCGA database. **(A)** Volcano plot of the differentially expressed lncRNAs between HNSCC tissues and adjacent tissues. The log2(fold change) on the X axis and—log10(*P*-value) on the Y axis are shown in the graph. Red dots represent high expression, and green dots represent low expression. Black shows lncRNA expression with both logFC < 1 and – log10 (*P*-value) < 0.05. Differentially expressed mRNAs and lncRNAs were calculated using edgeR, which identified upregulated overexpressed lncRNAs and 3,397 downregulated lncRNAs. **(B)** Venn diagram showing that eight DElncRNAs simultaneously associated with *HLA-A, HLA-B*, and *HLA-C*. **(C)** Kaplan-Meier survival curve of patients stratified according to low and high expression of *LINC02195* in the TCGA database (HNSCC). The median expression was used as the cut-off; *P* = 0.0180.

The TCGA database was used to investigate and analyze the expression of lncRNAs and mRNAs with the “edgeR” package. Notably, 5,731 upregulated DEmRNAs and DElncRNAs and 3,397 downregulated DEmRNAs and DElncRNAs were identified in HNSCC tissues (Figure 1A). Then, we extracted DElncRNA from the TCGA database and explored the correlations with the genes encoding MHC I molecules in humans (*HLA-A, HLA-B*, and *HLA-C*). The Venn diagram revealed eight DElncRNAs that were simultaneously associated with *HLA-A, HLA-B*, and *HLA-C*, namely, *PSMB8-AS1, TTLL11-IT1, LINC02195, LINC00623, LINC07871, LINC00944, LINC02574*, and *MIR3945HG* (Figure 1B and Supplementary Figure 1A, |Pearson's correlation coefficient| ≥ 0.4). According to the subsequent survival analysis, only *LINC02195* and *LINC01871* were correlated with patient prognosis (Figure 1C and Supplementary Figures 1B–H). As shown in Figure 1C, the Kaplan-Meier analysis revealed a significant correlation between high *LINC02195* expression and good prognoses for patients with HNSCC in the TCGA database (*P* = 0.0180). The median histoscore was used as the cut-off. The Cox proportional hazards model also showed an association between *LINC02195* expression and a good outcome (Table 1). Considering the correlation with the expression of MHC I molecules and prognostic value, *LINC02195* was selected for further research in this study.

**Table 1:** Multivariate analysis of the overall survival of patients with HNSCC based on TCGA.

**Parameters**	**HR (95%CI)**	***P*-value**
Sex	0.813 (0.594–1.113)	0.196
Age	1.021 (1.007–7.035)	0.003^*^
Pathological grade	1.047 (0.866–1.266)	0.637
Pathological stage	1.136 (0.964–1.338)	0.127
Tumor size	1.025 (0.901–1.164)	0.711
Node stage	1.125 (0.972–1.303)	0.115
*LINC02195* expression	0.743 (.0557–0.991)	0.043^*^

*Cox proportional hazards regression model*.

*HR hazard ratio, 95% CI 95% confidence interval*.

* *P < 0.05*.

### *LINC02195* Was Expressed at High Levels in HNSCC and Correlated With a Good Prognosis

**Figure 2:**
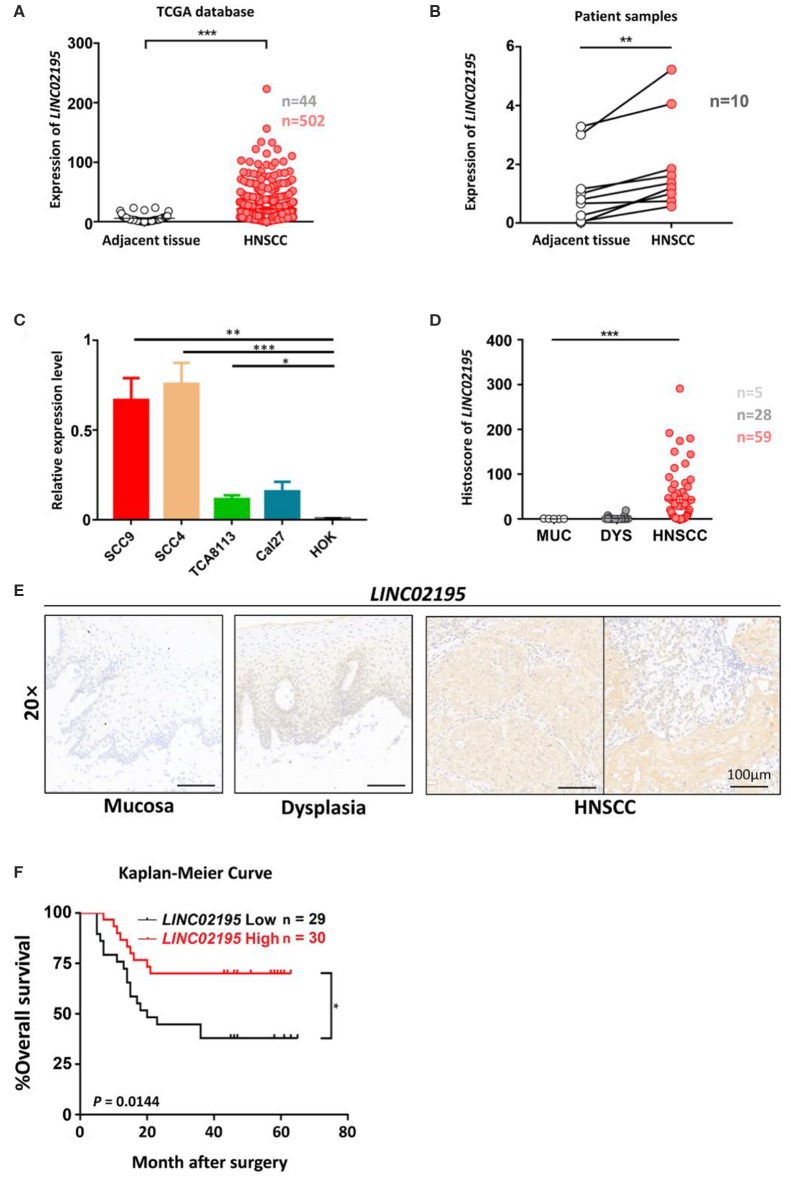
*LINC02195* was expressed at high levels in HNSCC tissues. **(A)** Relative expression level of *LINC02195* in HNSCC and adjacent tissues from TCGA database. ***P < 0.001. **(B)** Relative expression level of *LINC02195* in HNSCC and adjacent tissue samples from patients with HNSCC. **P < 0.01. **(C)** Relative expression level of *LINC02195* in 5 HNSCC cell lines (SCC9, SCC4, TCA8113, CAL27) and HOKs. *P < 0.05, **P < 0.01, and ***P < 0.001. **(D–E)** Statistical analysis **(F)** and representative images **(E)** of ISH using the probe for *LINC02195* in normal mucosal, dysplastic and tumor tissues. Scale bar, 100 μm. **(F)** Kaplan-Meier curve of patients stratified according to low and high expression of *LINC02195* in the cohort of the TMA. The median expression was used as the cut-off; *P* = 0.0144.

We next analyzed the expression and clinical features of *LINC02195* in patients with HNSCC. *LINC02195* was expressed at higher levels in tumor samples than in adjacent non-tumor tissues (Figure 2A, *P* < 0.001). Subsequently, 10 pairs of fresh HNSCC tissues matched with adjacent non-tumor tissues were used to verify the differential expression of *LINC02195*. Based on the qRT-PCR data, the expression in the HNSCC group was higher than that in the adjacent normal tissue group (Figure 2B, *P* = 0.0012). Then we explored the expression of *LINC02195* in human oral keratinocytes (HOKs) and the HNSCC cell lines SCC9, SCC4, TCA8113, and CAL27 using qRT-PCR. *LINC02195* was expressed at significantly higher levels in HNSCC cell lines than in HOKs (Figure 2C). Using ISH combined with a tissue microarray, *LINC02195* was expressed at higher levels in HNSCC tissues than in dysplastic tissue or the normal oral mucosa (Figures 2D,E, *P* < 0.001).

We analyzed the prognostic value of *LINC02195* and the relationship between clinicopathological data and *LINC02195* expression levels in the TCGA database and our patient cohort. The median histoscore was used as the cut-off. As shown in Figure 2F, the Kaplan-Meier analysis revealed a correlation between high *LINC02195* expression and a good prognosis for patients included in the TMA (*P* = 0.0144), consistent the result observed in TCGA. The Cox proportional hazards model also showed an association between *LINC02195* expression and a good outcome (Table 2).

**Table 2:** Multivariate analysis of overall survival in patients with primary HNSCC.

**Parameters**	**HR (95%CI)**	***P*-value**
Sex	0.866 (0.230–3.260)	0.832
Age	2.278 (0.905–5.731)	0.080
Pathological grade	1.333 (0.327–5.434)	0.688
Tumor size	1.363 (0.801–2.318)	0.254
Node stage	1.287 (0.767–2.162)	0.339
Smoking	0.850 (0.245–2.946)	0.798
Alcohol use	1.046 (0.349–3.134)	0.935
*LINC02195* expression	0.366 (0.140–0.956)	0.040^*^

*Cox proportional hazards regression model*.

*HR hazard ratio, 95% CI 95% confidence interval*.

* *P < 0.05*.

As shown in Table 3, *LINC02195* expression was not correlated with other clinical data in the TCGA database including age, sex, pathological grade, pathological stage, T classification, and N classification. One-way ANOVA and post-Tukey test of the human HNSCC tissue microarray data did not reveal associations of the *LINC02195* expression level with the pathological grade, T classification, N classification, recurrence, radiotherapy, lymphatic metastasis, smoking, or alcohol consumption (Supplementary Figure 2).

**Table 3:** Relationship between *LINC02195* expression and clinical parameters of patients in TCGA.

**Clinical parameter**	***n***	***LINC02195*** **expression in TCGA database**		
		**Mean ± SED**	***t***	***P***
Tumor vs. Normal				
Adjacent non-tumor tissue	44	5.68 ± 0.9405	4.123	<0.0001*
Tumor	502	21.66 ± 1.143		
Age (years)				
<60	245	21.73 ± 1.782	0.1238	0.9016
≥60	255	21.44 ± 1.458		
Sex				
Male	367	21.81 ± 1.354	0.2262	0.8211
Female	134	21.22 ± 2.149		
Pathological grade				
G1+G2	362	20.48 ± 1.3	1.402	0.1616
G3+G4	120	24.12 ± 2.234		
Pathological stage				
Stage I-II	95	22.93 ± 2.487	0.7006	0.4840
Stage III-IV	338	20.89 ± 1.377		
T				
T1+T2	178	22.58 ± 1.823	0.9838	0.3258
T2+T3	267	20.22 ± 1.538		
N				
N0	170	21.13 ± 1.793	0.2114	0.8327
N1+N2+N3	238	21.66 ± 1.706		

### *LINC02195* Was an Immune-Related lncRNA

**Figure 3:**
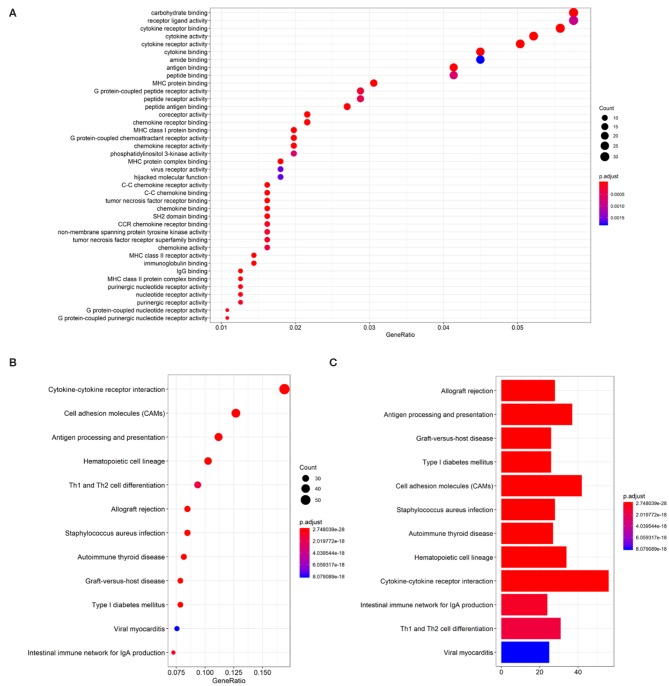
GO and KEGG pathway enrichment analysis for *LINC02195*-related mRNAs based on TCGA database. **(A)** Plot of the enriched GO terms associated with *LINC02195*-related mRNAs. **(B,C)** Plot of the KEGG pathways associated with *LINC02195*-related mRNAs. *P* < 0.05 and Cor > 0.4 were used as the thresholds to select GO and KEGG terms.

*LINC02195*-related mRNAs were selected for the gene annotation enrichment analysis to explore the biological functions of *LINC02195*. Sixty three enriched GO terms and 50 enriched KEGG pathways were associated with *LINC02195* (Figure 3 and Supplementary Table 1,2, *P* < 0.05). As shown in Figure 3A, the most significantly enriched GO terms were carbohydrate binding, receptor ligand activity, cytokine receptor binding and activity and cytokine binding and activity. Similarly, the significantly enriched pathways were cytokine-cytokine receptor interaction, cell adhesion molecules, antigen processing and presentation, hematopoietic cell lineage and Th1 and Th2 cell differentiation (Figures 3B,C). Interestingly, in terms of cytokine-cytokine receptor interactions and cell adhesion molecules, the GO analysis indicated that *LINC02195* was closely correlated with immune factors such as chemokines, class I and II helical cytokines and the T cell receptor signaling pathway (Supplementary Figure 3). Based on these findings, *LINC02195* was an immune-related lncRNA.

### *LINC02195* Silencing Decreased the Expression of MHC I Molecules in HNSCC

**Figure 4:**
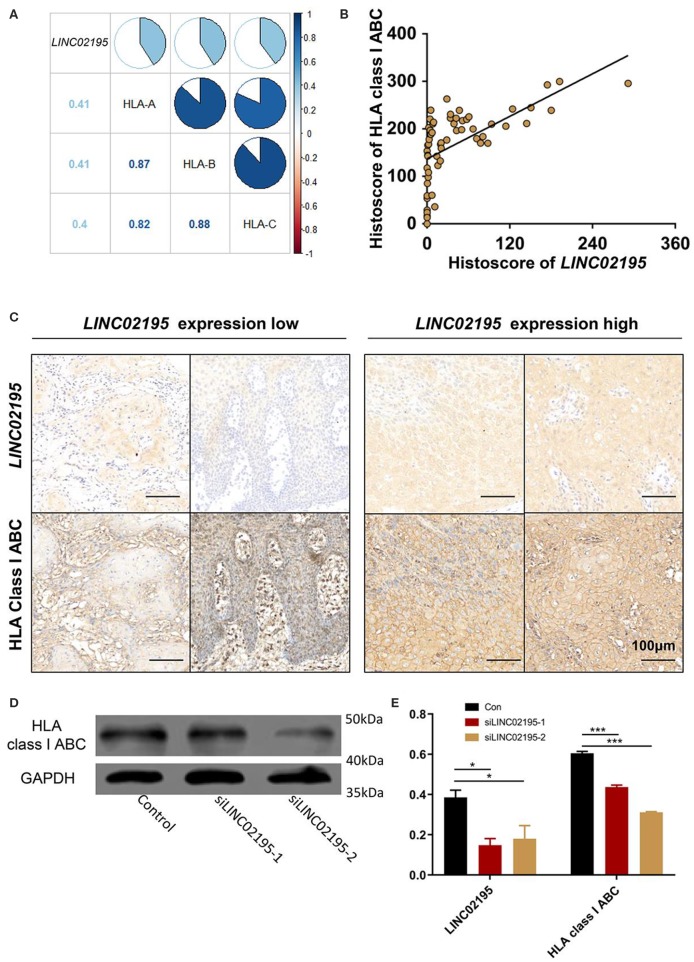
*LINC02195* was closely correlated with the expression of MHC I molecule in tumor cells. **(A)** Pearson's correlation coefficients among *LINC02195, HLA-A, HLA-B*, and *HLA-C* expression in HNSCC samples from TCGA database. **(B)** Pearson's correlation coefficients between the histoscore of *LINC02195* and HLA class I ABC in the human HNSCC TMA; *P* < 0.0001, *r* = 0.6103. **(C)** Representative images of ISH and IHC for *LINC02195* and HLA class I ABC in human HNSCC specimens (low *LINC02195* expression vs. high *LIN02195* expression). Scale bar: 100 μm. **(D,E)** *in vitro, LINC02195* knockdown by using siLINC02195 downregulated the expression of HLA class I ABC in the SCC9 cell line. *P < 0.05 and ***P < 0.001.

The GO enrichment analysis also revealed a close association of *LINC02195* with MHC I protein binding (GO:0042288, *P* = 1.67E−12, Supplementary Table 1). Moreover, KEGG pathway analyses identified close associations of *LINC02195* with antigen processing and presentation (hsa04612, *P* = 3.00E−30, Supplementary Table 2). As shown in Figure 4A, *LINC02195* was closely correlated with the expression of *HLA-A, HLA-B*, and *HLA-C*, which encode classical MHC I molecules. Next, using an anti-HLA ABC antibody, the significant correlation between *LINC02195* and MHC I was confirmed in our patient cohort (Figures 4B,C, *P* < 0.001). *In vitro*, si*LINC02195* was used to silence the expression of *LINC02195*, and HLA-A, -B, and -C expression was downregulated when *LINC02195* was silenced (Figures 4D,E, *P* < 0.001).

### *LINC02195* Correlated With CD8^+^ and CD4^+^ T Cell Infiltration in the HNSCC Tumor Microenvironment (TME)

**Figure 5:**
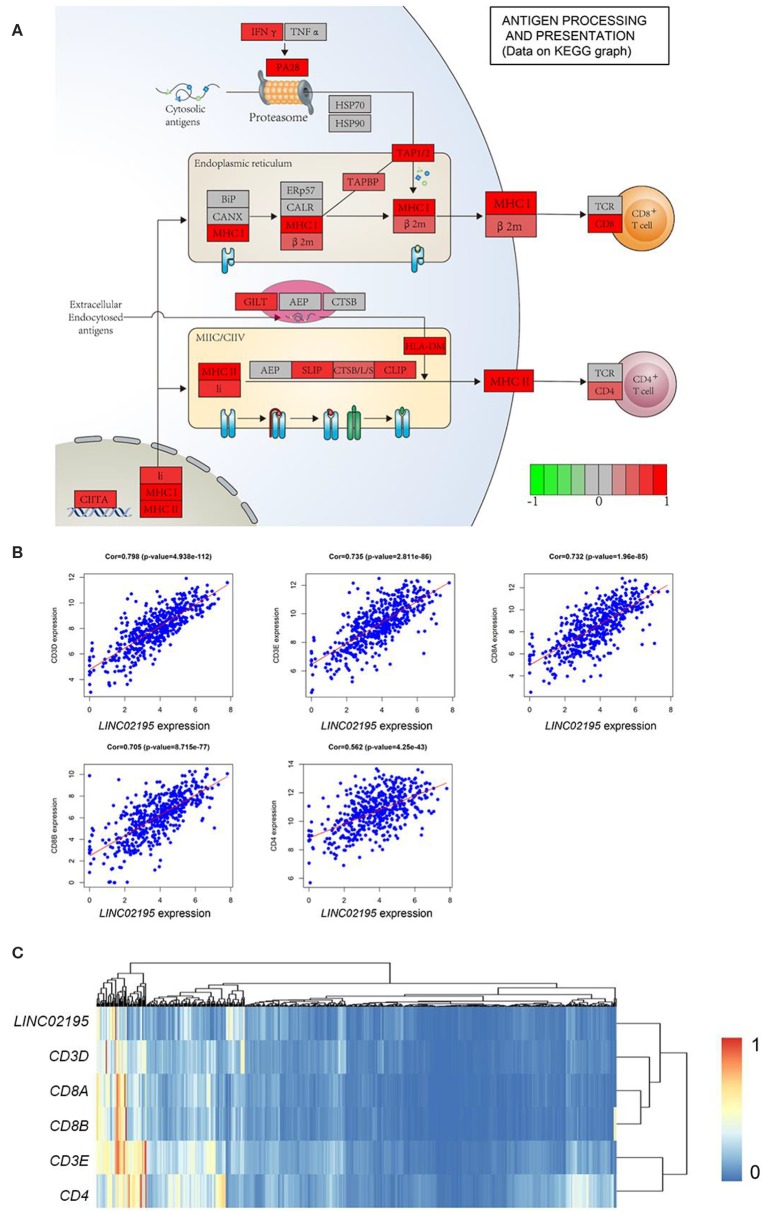
*LINC02195* expression was positively correlated with genes involved in antigen processing and presentation. **(A)** KEGG pathway enrichment analyses showed a close correlation between *LINC02195* expression and antigen processing and presentation in both the MHC I and II pathways. **(B)** Pearson's correlation coefficients between *LINC02195* and *CD3D, CD3E, CD8A, CD8B*, or *CD4* gene expression based on TCGA data. **(C)** Hierarchical clustering analysis of *LINC02195, CD3D, CD3E, CD8A, CD8B*, and *CD4* in TCGA database.

According to the KEGG pathway analysis, *LINC02195* was closely correlated with genes that are important for antigen processing and presentation (Figure 5A). T cells in the TME, which detect the antigens presented by MHC molecules, have been reported to be associated with good clinical outcomes in patients with many types of cancer, including bladder, breast, ovarian, colorectal, and renal cancers (25, 26). The correlation between MHC I and *LINC02195* expression was previously proven. Additionally, the correlation between *LINC02195* and MHC II molecules was also highly significant (Supplementary Figure 4). Therefore, the correlation between *LINC02195* and T cells in TME was analyzed. By performing a bioinformatics analysis, we observed close correlations of *LINC02195* expression with *CD3D, CD3E, CD8A, CD8B*, and *CD4* expression in the TCGA database (Figures 5B,C). Pearson's correlation analysis revealed a strong association between *LINC02195* and T cells in the TME. Based on the hierarchical clustering analysis, *LINC02195* was more relevant to CD8^+^ T cells than to CD4^+^ T cells.

**Figure 6:**
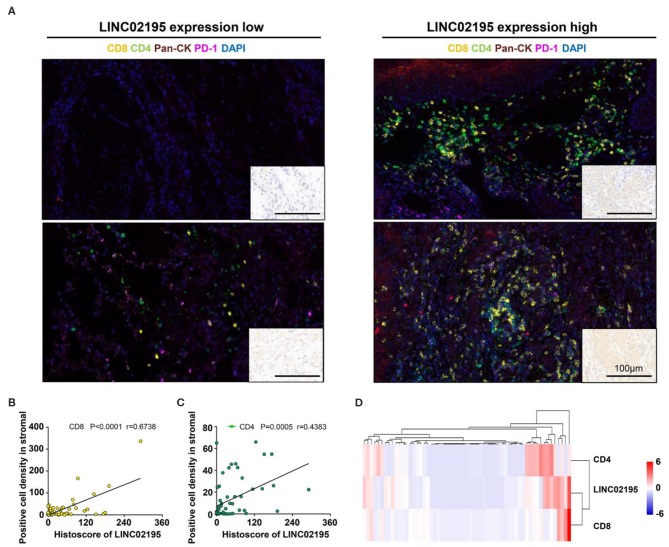
LINC02195 expression was positively correlated with the infiltration of CD8^+^ and CD4^+^ T cells in the TME. **(A)** Representative image of multiplex staining (CD8, CD4, PD-1, pan-keratin, and DAPI) in human HNSCC specimens (low *LINC02195* expression vs. high *LINC02195* expression). Scale bar, 100 μm. **(B,C)** Pearson's correlation coefficient between the histoscore of *LINC02195* and the number of CD8^+^ or CD4^+^ cells in the human HNSCC TMA. **(D)** Hierarchical clustering analysis of *LINC02195*, CD4, and CD8 expression in human HNSCC samples (*n* = 59).

Multiplex IHC was performed to analyze the immune status in the TME and verify this finding. As shown in Figure 6, tumor tissues with high *LINC02195* expression exhibited more infiltrating CD8^+^ and CD4^+^ T cells than tumor tissues with low *LINC02195* expression (Figure 6A). Interestingly, Pearson's correlation analysis identified a positive correlation between the increase in *LINC02195* expression and the number of infiltrating CD8^+^ and CD4^+^ T cells (Figures 6B,C; CD8, *P* < 0.0001; CD4, *P* = 0.0005). The hierarchical clustering analysis further clarified the relationship among these molecules (Figure 6D).

## Discussion

In this study, the expression of the lncRNA *LINC02195* was closely associated with MHC molecules. Moreover, it was expressed at high levels in human HNSCC tissues, based on the analyses of TCGA database and patient samples. In addition, patients with high *LINC02195* expression exhibited better prognoses than patients with low *LINC02195* expression. We conducted GO and KEGG enrichment analyses to clarify the function and protein-protein interaction network of *LINC02195* by identifying *LINC02195*-related mRNAs in HNSCC tissues in the TCGA database and to determine the explanation for this outcome. *LINC02195* expression was closely correlated with the expression of MHC I molecules. Through further western blotting and immunostaining analysis, the correlation was further verified *in vitro*. Multiplex staining and Pearson's correlation analyses were used to verify the significant relationship between the lncRNA and infiltrating CD8^+^ and CD4^+^ T cells in TME.

LncRNAs have been widely investigated in recent years. An increasing number of lncRNAs have been shown to regulate the immune system (27). *LINC02195*, a newly annotated lncRNA, is located on chromosome 16p12.1. In normal tissues, it is expressed at high levels in the appendix, esophagus, lymph node, stomach, thyroid, some immune cell-rich places in our body, which suggest it may correlate with immune function (28). However, the relationship between *LINC02195* and HNSCC has not been revealed. In this study, *LINC02195* was confirmed to be associated with HNSCC by analyzing HNSCC and normal tissues. We found *LINC02195* is mostly found in the nucleus and cytoplasm by ISH. Moreover, high expression of this lncRNA was associated with a good prognosis for patients with HNSCC. GO and KEGG analyses revealed that *LINC02195* was an immune-related lncRNA. This lncRNA was correlated with antigen processing and presentation. MHC I molecules have a crucial role in this process, and previous studies have reported an association between the expression of the lncRNA *HCP5* expression was associated with *HLA-B* expression (19). In the present study, the Pearson's correlation analysis indicated a close correlation between genes encoding MHC I molecules with *LINC02195*. Moreover, western blotting revealed a decrease in MHC I levels upon the silencing of *LINC02195*. The results suggested that *LINC02195* may interact with related enzymes or molecules encoding MHC I in the nucleus and further regulating the expression of MHC I molecules.

In recent years, the TME has attracted increasing attention from researchers because of its close correlation with prognosis and treatment. A number of various types of immune cells infiltrate the TME (25). Substantial T cell infiltration is associated with good clinical outcomes and positive responses to immunotherapy in many types of cancer (25, 29). However, the relationship between lncRNAs and T cells in the TME remains unclear. As shown in a previous study, *lnc-EGFR* stimulates Treg differentiation and subsequently promotes the immune evasion of hepatocellular cancer (18). According to another study, a lncRNA, *LINK-A*, downregulated cancer cell antigen presentation and was negatively correlated with T cells in the TME of triple-negative breast cancer (30). In the present study, gene enrichment and pathway analyses identified a strong correlation between *LINC02195* and T cells in the tumor context. Peptide–MHC I complexes are transported to the plasma membrane for cancer cell antigen presentation to CD8^+^ T cells, which attack malignant cells. Next, through multiplex IHC of the human HNSCC TMA, we observed a positive correlation between *LINC02195* expression and the density of CD8^+^ and CD4^+^ T cells in the TME.

In summary, this study is the first to explore the role of *LINC02195* in HNSCC. High *LINC02195* expression were correlated with a good prognosis for patients with HNSCC. *LINC02195* may play a fundamental role in regulating the expression of MHC I molecules. In addition, *LINC02195* was an immune-related lncRNA and positively correlated with an increased T cell density. This evidence, therefore, supports the hypothesis that *LINC02195* is a promising prognostic marker for HNSCC and a target for future therapeutic interventions.

## Data Availability Statement

The raw data supporting the conclusions of this article will be made available by the authors, without undue reservation, to any qualified researcher.

## Author Contributions

HL and H-GX contributed to conception, design, data acquisition, analysis, drafted, and critically revised the manuscript. YX, Q-CY, S-CY, and H-CT contributed to data acquisition, drafted and critically revised the manuscript. W-FZ and Z-JS contributed to conception, data analysis, and interpretation, drafted and critically revised the manuscript. All authors provided final approval and agreed to be accountable for all aspects of the study.

## Conflict of Interest

The authors declare that the research was conducted in the absence of any commercial or financial relationships that could be construed as a potential conflict of interest.
