# Supplementary material for: Long Non-coding RNA LINC02195 as a Regulator of MHC I Molecules and Favorable Prognostic Marker for Head and Neck Squamous Cell Carcinoma
Source: Front Oncol. 2020 May 6;10:615. doi: 10.3389/fonc.2020.00615 (PMC7218046; doi:10.3389/fonc.2020.00615)
Supplement: Supplementary file 1 [file Data_Sheet_1.DOCX]

Supplementary Material

**Supplementary Materials and Methods**

**Study Population and RNA expression data for bioinformatic analyses**

The RNA expression data HNSCC cases including 502 HNSCC tumor samples and 44 normal tissue samples were downloaded from the TCGA database derived from the data portal (https://gdc.cancer.gov/). The data included the expression of RNA (mRNA and noncoding RNA) (level 3) and patient clinical data of 546. The RNA expression data was processed and analyzed by Python and R language.

**Identification of DEIncRNAs and DEmRNAs**

mRNAs and lncRNAs were identified by using the Ensembl database. Before conducting differential expression analysis, we ruled out all unexpressed RNAs by removing all rows with a mean read of less than or equal to one. The differentially expressed lncRNAs (DElncRNAs) and mRNAs (DEmRNAs) were found by using the using “edgeR” package, a bioconductor package via R language procedure. All P-values used false discovery rate (FDR) to correct the statistical significance of the multiple test. |FoldChange| ≧2 and FDR < 0.01 were considered significant. For the obtained DEIncRNAs and DEmRNAs, we generated volcano plot using the “ggplot2” packages in R language.

**Gene enrichment and functional annotation analysis**

The q value of mRNAs which were greater or equal to 0.4 were involved in further function enrichment analysis. And the bubble map was drawn using the "ggplot2" R package. The mRNAs with significant value of the Pearson correlation coefficient (|Pearson correlation coefficient| ≥ 0.4) were included in further function enrichment analysis. The Gene Ontology (GO) and Kyoto Encyclopedia of Genes and Genomes (KEGG) analyses were performed by R language software using the “clusterProfiler” package. The enriched GO terms and KEGG pathways with P < 0.05 were included in *LINC02195*-related biologic processes or signaling pathways. Hierarchical clustering was performed by “pheatmap” packaged in R language.

**Human HNSCC Tissue sample**

The study was approved by the Ethics Committee of the School and Hospital of Stomatology of Wuhan University Medical Ethics Committee. The patient cohort included 5 patients with normal oral mucosae, 28 with oral epithelial dysplasia and 59 with HNSCC and 10 paired fresh HNSCC samples. The clinical features including TNM classification, histological grade and overall survival were available for all cases. The clinical TNM classification of the HNSCC was classified according to the guidelines of the American Joint Committee on Cancer (8th edition). The clinicopathologic characteristics and follow-up data for these patients were retrieved from the electronic medical records. Two independent pathologists marked the tumor areas of each tissue on hematoxylin and eosin-stained slides and diagnosed each specimen. Then the tissues were assembled into tissue microarray (TMA).

**In Situ Hybridization (ISH), Immunohistochemistry (IHC) and Histological Quantification**

To examine the expression of *LINC02195*, ISH was used on human HNSCC TMA with digoxigenin-labeled antisense oligonucleotide probes as previously described[[21](#_ENREF_21)]. The probe sequences for *LINC02195* were as follows: 5‘-DIG-TCCTTTGGAATCCTCCTACTTTGGCAGC-3’. IHC staining was performed as described[[22](#_ENREF_22)]. Signals were detected using biotinylated goat anti-rabbit or anti-mouse followed by streptavidin HRP. Staining was visualized with DAB (Dako) and counterstained with hematoxylin (Dako), sealed with neutral resins, and photographed. The scanning of TMA and processing of histoscore were acquired as previously described[[22](#_ENREF_22)]. Human leukocyte antigen (HLA) class I ABC antibody (1:300, proteintech, USA) was used to detect MHC I molecules in human HNSCC samples.

**Cell lines, siRNAs and western blotting**

The cell lines SCC4, SCC9 and Cal27 were obtained from the ATCC (American Type Culture Collection). Tca8113 were acquired from the Ninth People’s Hospital, Shanghai Jiao Tong University School of Medicine. Human oral keratinocyte line (HOK) were obtained from ScienCell. Cal27, Tca8113 and HOK were maintained in DMEM/high glucose supplemented with 10% FBS (Gibco, Grand Island, USA) and 1% streptomycin-penicillin (Gibco) at 37℃ under 5% CO_2_ in chamber. SCC4 and SCC9 was cultured in DMEM/F-12 with 10% FBS (Gibco, NY, USA) and 1% streptomycin-penicillin.

Small interfering RNAs (siRNAs) targeting *LINC02195* were designed and synthesized by GenePharma (Suzhou, China). SCC9 cells were transfected with the siRNAs using Lipofectamine™ 3000 (Life Technologies, USA) according to the manufacturer’s instructions. The following sequences were used: si*LINC02195*-1, 5′-UCAACACUCAUCGGUGUAUTT-3′ (sense), 5′-AUACACCGAUGAGUGUUGATT -3′ (antisense), si*LINC02195*-2 , 5′- GCAACCAGAAGUUCAUCCATT-3′ (sense), 5′-UGGAUGAACUUCUGGUUGCTT -3′ (antisense),and negative control (si-NC), 5′-UUCUCCGAACGUGUCACGUTT-3′ (sense), 5′-ACGUGACACGUUCGGAGAATT-3′ (antisense).

Western blotting with whole-cell protein extracts from SCC9 cells were performed as described[[21](#_ENREF_21)]. Antibodies for western blot: HLA class I ABC antibody (15240-1-AP; proteintech, USA). GAPDH protein levels were used as loading controls. The experiments were repeated three times.

**Total RNA extraction and qRT-PCR analysis**

Total RNA extraction and qRT-PCR analysis have been described previously[[21](#_ENREF_21)]. The *LINC02195* expression were calculated with the comparative Ct method (2^−ΔΔCT^) and normalized with GAPDH expression. All experiments were performed in triplicate. The following primers were used: GAPDH-F: 5’-TGATGACCCTTTTGGCTCCC-3’, GAPDH-R: 5’-GGAAGCTTGTCATCAATGGAAATC-3’; *LINC02195*-F: 5’-GGTCCTTTGGAATCCTCCTACTTT-3’, *LINC02195*-R: 5’-CTTTCTGCGTCATCGGTGTCTT-3’.

**Multiplex IHC and image analysis**

Opal™ 7-Color Manual IHC Kit (NEL811001KT; PerkinElmer, Hopkinton, MA,USA) was used to stain the TMA. In brief, the slide was deparaffinized in xylene, rehydrated, and washed in tap water before boiling in 1X AR6 buffer (pH 6; PerkinElmer, Hopkinton, MA,USA) for microwave treatment (MWT) in order to retrieve epitope. Then, the slide was blocked using Blocking/Ab Diluent (PerkinElmer) and incubated with primary antibody for 1h at room temperature. Primary antibodies included CD4 (1:1000; Abcam, USA), Pan-Keratin (1:350; Cell Signaling Technology, USA), CD8a (1:1000; Cell Signaling Technology), PD-1 (1:500; Cell Signaling Technology). After washing in TBST wash buffer for 5 mins, incubation with Opal™ polymer HRP Ms + Rb (PerkinElmer) was performed at room temperature for 30 min. Drain off excess wash buffer and pipette 100-300 µL of Opal Fluorophore Working Solution (PerkinElmer) onto the slide. Incubate the slide at room temperature for 10 mins. Multiplex TSA was optimized by performing a duplex (CD4, Opal 620 and CD8, Opal 570), followed by a triplex (addition of PD-1, Opal 690), 4plex (addition of pan-keratin, Opal 520). All multiplex TSA experiments were performed by repeating staining cycles in series, with MWTs in between each cycle and at the end of the multiplex TSA. After all multiplex TSA stainings finished, the slide was stained with a DAPI and were enclosed.

Slides were scanned using the PerkinElmer Vectra (PerkinElmer). Multispectral images were unmixed using spectral libraries built from images of single stained tissues for each reagent using the inform Advanced Image Analysis software (inForm 2.1.1; PerkinElmer). A selection of 15–25 representative original multispectral images was used to train the inForm software (tissue segmentation, cell segmentation, phenotyping tool, and positivity score). The tumor and stromal area were distinguished and calculated by inForm software using pan-keratin as tumor marker. With this data, the positive cell density was subsequently generated by the following formula: positive cell density = the number of positive cells in stroma ÷ the area of stroma (calculating by image pixels).

**Statistical Analysis**

All data are presented as the mean values ± SEM. The statistical analyses were performed by Prism 7.00 (GraphPad Software Inc., USA). Statistical significance was assessed by the one-way ANOVA followed by Tukey multiple comparison test and t-test. Kaplan-Meier curves followed by log-rank test were used to analyze the survival of patient. The Cox proportional hazards model was used for multivariate analysis to assess the significance of overall differences. The statistical significance level for all comparisons was set at P < 0.05. *, **, *** represent P < 0.05, P < 0.01, and P < 0.001, respectively.

**Supplemantary Figure 1**


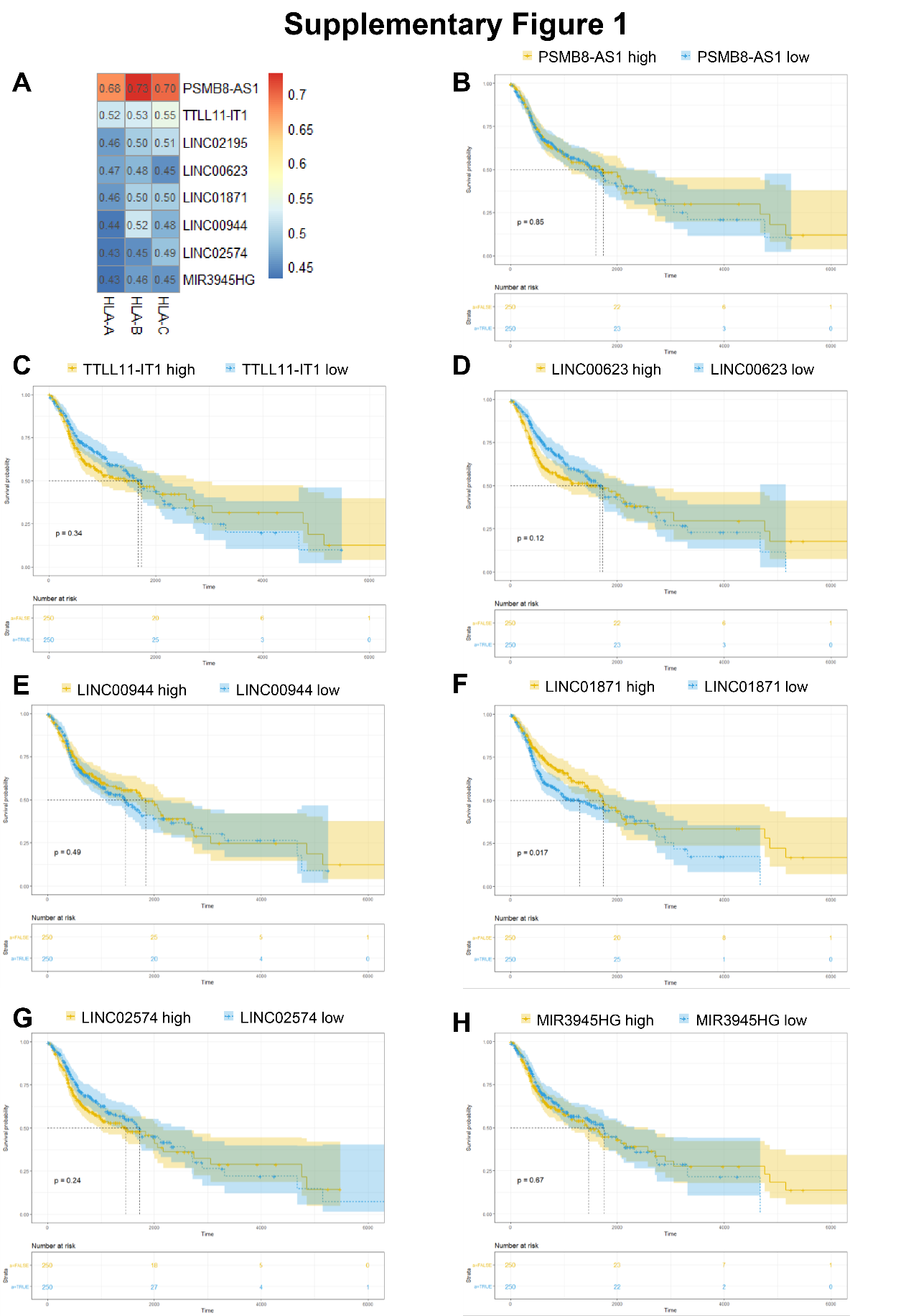


**Supplementary Figure 1 Kaplan-Meier survival curves of lncRNAs which correlated with MHC class I molecules.**

**A** The heat map showed that *LINC02195* was the most significant DElncRNA among the six lncRNAs. The |Pearson correlation coefficient| among lncRNAs, HLA-A, HLA-B and HLA-C.

**B-H** Kaplan-Meier survival curves according to low and high expression of *PSMB8-AS1, TTLL11-IT1, LINC00623,* *LINC00944, LINC01871, LINC02574 and MIR3945HG* in TCGA database. The median of expression was used as cut-off.

**Supplemantary Figure 2**


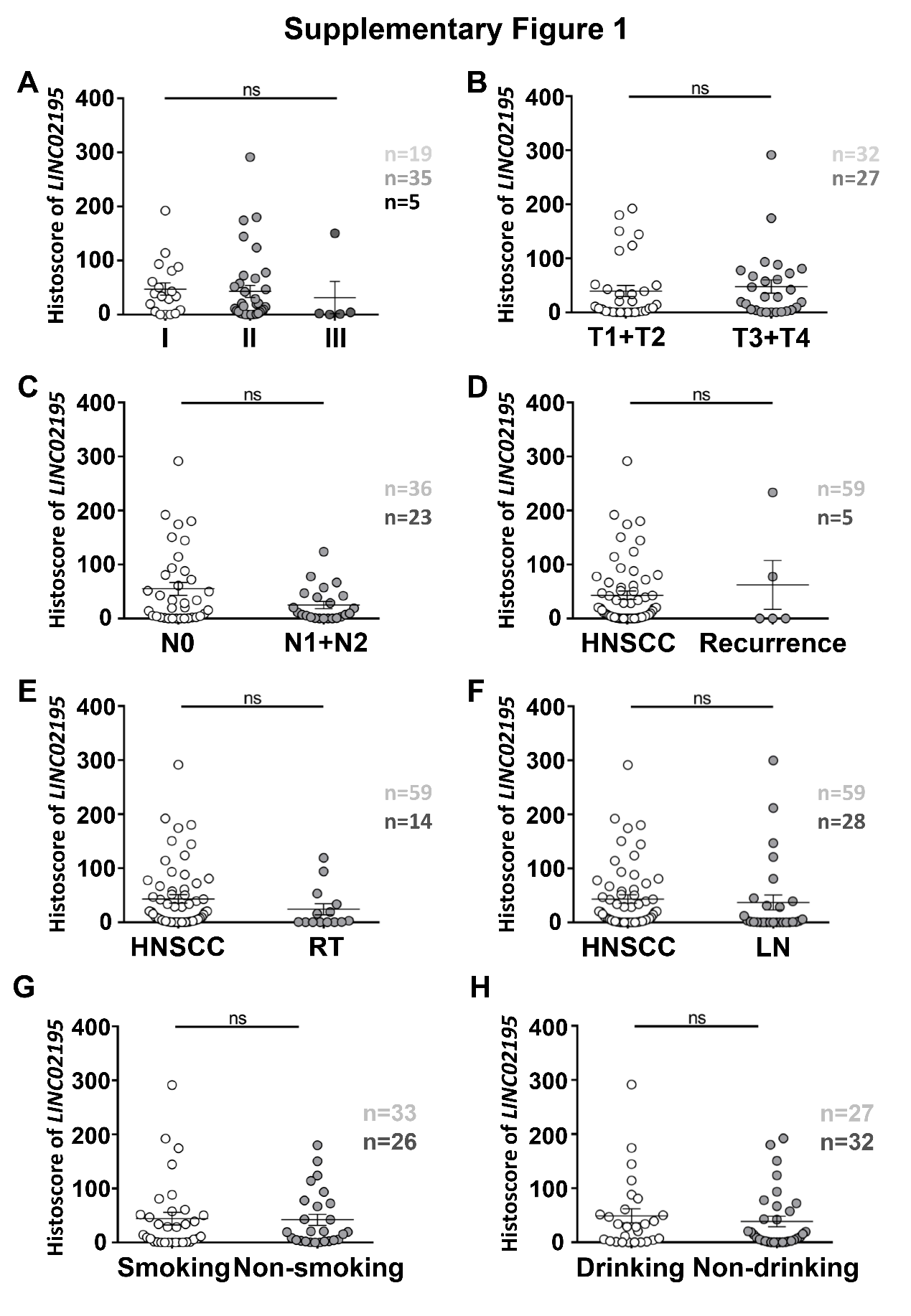


**Supplementary Figure 2 *LINC02195* expression in other clinical parameters.**

**A** Quantification of the histoscores of *LINC02195* by pathological grade (I, II and III); P > 0.05.

**B** Quantification of histoscores of *LINC02195* between T1+ T2 and T3+ T4; P > 0.05.

**C** Quantification of histoscores of *LINC02195* between N1 and N2+N3; P > 0.05.

**D** Quantification of histoscores of *LINC02195* in primary HNSCC compared with recurrent HNSCC; P > 0.05.

**E** Quantification of the immunohistochemical histoscores of *LINC02195* in primary HNSCC compared with preoperative radiotherapy; P > 0.05.

**F** Quantification of the immunohistochemical histoscores of *LINC02195* in primary HNSCC compared with metastatic lymph node tissue; P > 0.05.

**G** Quantification of the histoscores of *LINC02195* in smoker compared non-smoker; P > 0.05.

**H** Quantification of the histoscores of *LINC02195* in drinker compared non-drinker; P > 0.05.

All data are presented as the means ± SEM.


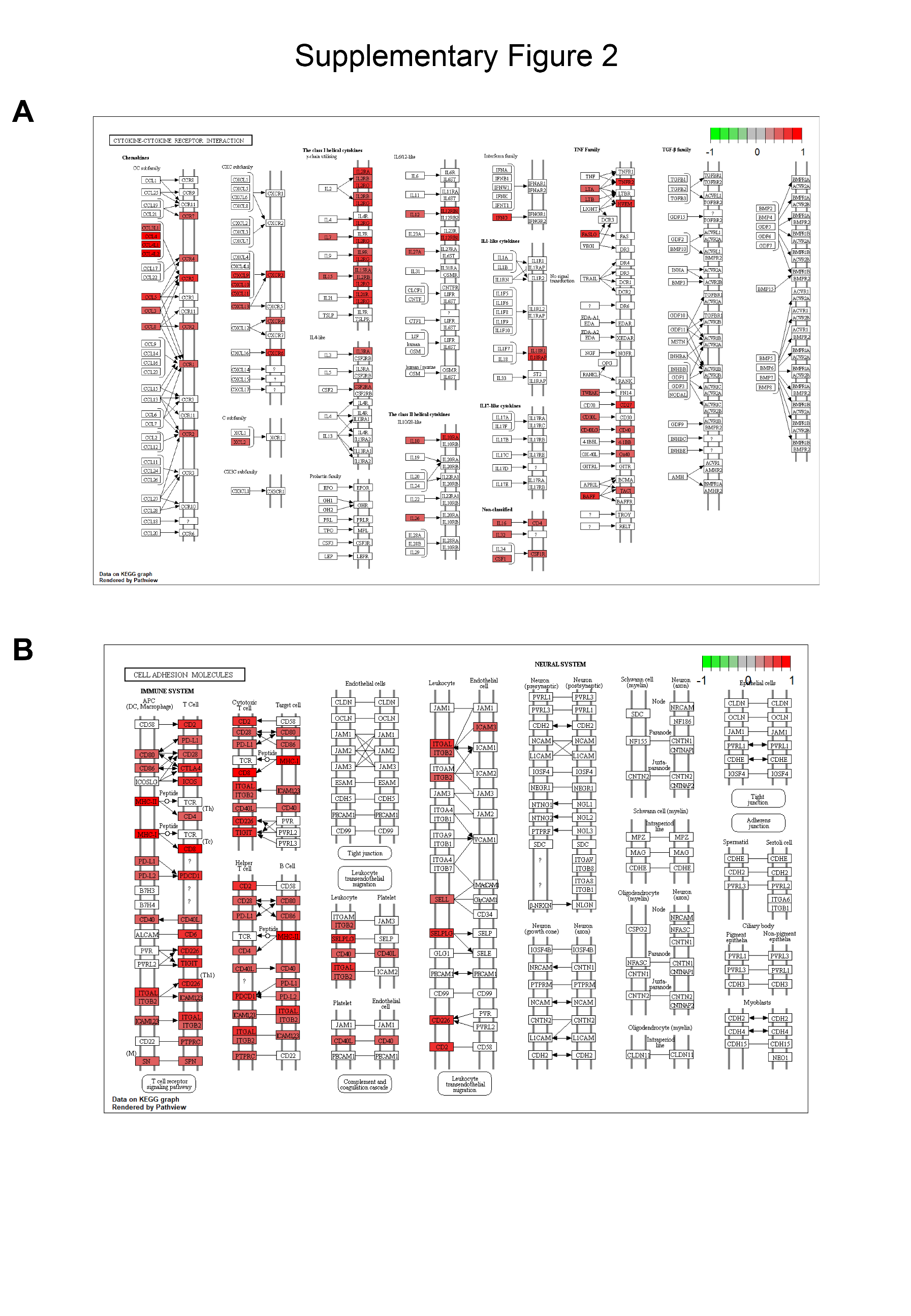
**Supplemantary Figure 3**

**Supplementary Figure 3 KEGG pathway analysis indicated that *LINC02195* related with cytokine-cytokine receptor interaction and cell adhesion molecules.**

**A** KEGG pathway analysis showed that *LINC02195* correlated with cytokine-cytokine receptor interaction especially with chemokines and the class I helical cytokines.

**B** KEGG pathway analysis showed that *LINC02195* positively correlated with cell adhesion molecules especially with T cell receptor signaling pathway.

**Supplemantary Figure 4**


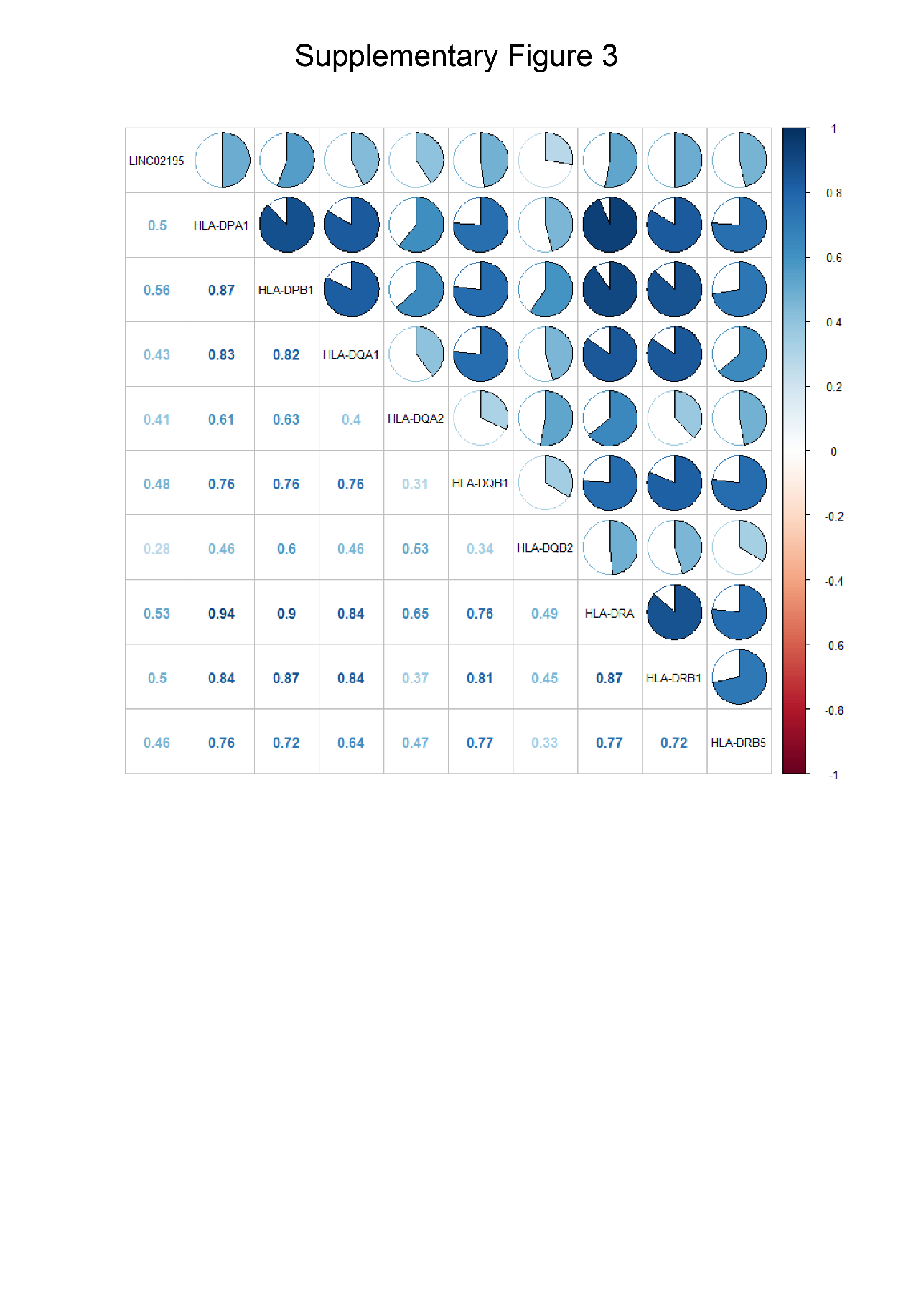


**Supplementary Figure 4 *LINC02195* expression correlated with HLA genes which encoding MHC class II proteins.**

**Supplementary Table 1** GO enrichment analysis of LINC02195 in TCGA database (HNSCC).

| ID | Description | GeneRatio | BgRatio | pvalue | p.adjust | qvalue | Count |
| --- | --- | --- | --- | --- | --- | --- | --- |
| GO:0004896 | cytokine receptor activity | 28/556 | 92/17632 | 2.32E-20 | 1.37E-17 | 1.19E-17 | 28 |
| GO:0042287 | MHC protein binding | 17/556 | 36/17632 | 1.17E-16 | 3.47E-14 | 3.00E-14 | 17 |
| GO:0042605 | peptide antigen binding | 15/556 | 30/17632 | 2.52E-15 | 4.99E-13 | 4.32E-13 | 15 |
| GO:0019955 | cytokine binding | 25/556 | 125/17632 | 1.19E-13 | 1.76E-11 | 1.52E-11 | 25 |
| GO:0042288 | MHC class I protein binding | 11/556 | 19/17632 | 1.67E-12 | 1.98E-10 | 1.72E-10 | 11 |
| GO:0032395 | MHC class II receptor activity | 8/556 | 10/17632 | 3.96E-11 | 3.91E-09 | 3.39E-09 | 8 |
| GO:0005125 | cytokine activity | 29/556 | 219/17632 | 6.43E-11 | 5.45E-09 | 4.72E-09 | 29 |
| GO:0030246 | carbohydrate binding | 32/556 | 269/17632 | 1.13E-10 | 7.54E-09 | 6.53E-09 | 32 |
| GO:0023023 | MHC protein complex binding | 10/556 | 20/17632 | 1.25E-10 | 7.54E-09 | 6.53E-09 | 10 |
| GO:0001637 | G protein-coupled chemoattractant receptor activity | 11/556 | 26/17632 | 1.40E-10 | 7.54E-09 | 6.53E-09 | 11 |
| GO:0004950 | chemokine receptor activity | 11/556 | 26/17632 | 1.40E-10 | 7.54E-09 | 6.53E-09 | 11 |
| GO:0005126 | cytokine receptor binding | 31/556 | 281/17632 | 1.45E-09 | 7.15E-08 | 6.19E-08 | 31 |
| GO:0015026 | coreceptor activity | 12/556 | 42/17632 | 4.01E-09 | 1.83E-07 | 1.58E-07 | 12 |
| GO:0019864 | IgG binding | 7/556 | 11/17632 | 8.83E-09 | 3.74E-07 | 3.24E-07 | 7 |
| GO:0016493 | C-C chemokine receptor activity | 9/556 | 23/17632 | 1.59E-08 | 6.29E-07 | 5.45E-07 | 9 |
| GO:0019957 | C-C chemokine binding | 9/556 | 24/17632 | 2.48E-08 | 9.18E-07 | 7.95E-07 | 9 |
| GO:0003823 | antigen binding | 23/556 | 199/17632 | 8.24E-08 | 2.87E-06 | 2.49E-06 | 23 |
| GO:0023026 | MHC class II protein complex binding | 7/556 | 16/17632 | 2.67E-07 | 8.79E-06 | 7.61E-06 | 7 |
| GO:0019865 | immunoglobulin binding | 8/556 | 23/17632 | 3.00E-07 | 9.29E-06 | 8.05E-06 | 8 |
| GO:0005164 | tumor necrosis factor receptor binding | 9/556 | 31/17632 | 3.13E-07 | 9.29E-06 | 8.05E-06 | 9 |
| GO:0019956 | chemokine binding | 9/556 | 32/17632 | 4.24E-07 | 1.20E-05 | 1.04E-05 | 9 |
| GO:0042169 | SH2 domain binding | 9/556 | 33/17632 | 5.67E-07 | 1.53E-05 | 1.32E-05 | 9 |
| GO:0042379 | chemokine receptor binding | 12/556 | 66/17632 | 8.95E-07 | 2.31E-05 | 2.00E-05 | 12 |
| GO:0001608 | G protein-coupled nucleotide receptor activity | 6/556 | 14/17632 | 2.32E-06 | 5.19E-05 | 4.50E-05 | 6 |
| GO:0045028 | G protein-coupled purinergic nucleotide receptor activity | 6/556 | 14/17632 | 2.32E-06 | 5.19E-05 | 4.50E-05 | 6 |
| GO:0001614 | purinergic nucleotide receptor activity | 7/556 | 21/17632 | 2.36E-06 | 5.19E-05 | 4.50E-05 | 7 |
| GO:0016502 | nucleotide receptor activity | 7/556 | 21/17632 | 2.36E-06 | 5.19E-05 | 4.50E-05 | 7 |
| GO:0048020 | CCR chemokine receptor binding | 9/556 | 43/17632 | 6.25E-06 | 0.000132 | 0.000115 | 9 |
| GO:0035586 | purinergic receptor activity | 7/556 | 25/17632 | 8.75E-06 | 0.000179 | 0.000155 | 7 |
| GO:0004715 | non-membrane spanning protein tyrosine kinase activity | 9/556 | 46/17632 | 1.12E-05 | 0.000215 | 0.000186 | 9 |
| GO:0032813 | tumor necrosis factor receptor superfamily binding | 9/556 | 46/17632 | 1.12E-05 | 0.000215 | 0.000186 | 9 |
| GO:0008528 | G protein-coupled peptide receptor activity | 16/556 | 145/17632 | 1.39E-05 | 0.000258 | 0.000224 | 16 |
| GO:0008009 | chemokine activity | 9/556 | 49/17632 | 1.93E-05 | 0.000346 | 0.0003 | 9 |
| GO:0001653 | peptide receptor activity | 16/556 | 151/17632 | 2.32E-05 | 0.000405 | 0.000351 | 16 |
| GO:0035004 | phosphatidylinositol 3-kinase activity | 11/556 | 79/17632 | 3.60E-05 | 0.00061 | 0.000528 | 11 |
| GO:0042277 | peptide binding | 23/556 | 285/17632 | 3.86E-05 | 0.000635 | 0.00055 | 23 |
| GO:0048018 | receptor ligand activity | 32/556 | 478/17632 | 5.52E-05 | 0.000884 | 0.000766 | 32 |
| GO:0001618 | virus receptor activity | 10/556 | 74/17632 | 0.000105 | 0.001596 | 0.001383 | 10 |
| GO:0104005 | hijacked molecular function | 10/556 | 74/17632 | 0.000105 | 0.001596 | 0.001383 | 10 |
| GO:0033218 | amide binding | 25/556 | 348/17632 | 0.00012 | 0.001777 | 0.001539 | 25 |
| GO:0017124 | SH3 domain binding | 13/556 | 127/17632 | 0.000189 | 0.002733 | 0.002367 | 13 |
| GO:0035591 | signaling adaptor activity | 10/556 | 80/17632 | 0.000203 | 0.002869 | 0.002486 | 10 |
| GO:0001784 | phosphotyrosine residue binding | 7/556 | 40/17632 | 0.000225 | 0.003106 | 0.002691 | 7 |
| GO:0005070 | SH3/SH2 adaptor activity | 8/556 | 54/17632 | 0.000269 | 0.003501 | 0.003033 | 8 |
| GO:0045236 | CXCR chemokine receptor binding | 4/556 | 11/17632 | 0.000271 | 0.003501 | 0.003033 | 4 |
| GO:0046934 | phosphatidylinositol-4,5-bisphosphate 3-kinase activity | 9/556 | 68/17632 | 0.000272 | 0.003501 | 0.003033 | 9 |
| GO:0019239 | deaminase activity | 6/556 | 31/17632 | 0.00036 | 0.004538 | 0.003931 | 6 |
| GO:0052813 | phosphatidylinositol bisphosphate kinase activity | 9/556 | 71/17632 | 0.000378 | 0.004665 | 0.004041 | 9 |
| GO:0004126 | cytidine deaminase activity | 4/556 | 12/17632 | 0.000396 | 0.004788 | 0.004148 | 4 |
| GO:0016814 | hydrolase activity, acting on carbon-nitrogen (but not peptide) bonds, in cyclic amidines | 6/556 | 34/17632 | 0.000606 | 0.007192 | 0.00623 | 6 |
| GO:0005085 | guanyl-nucleotide exchange factor activity | 22/556 | 327/17632 | 0.000733 | 0.008528 | 0.007388 | 22 |
| GO:0046935 | 1-phosphatidylinositol-3-kinase regulator activity | 4/556 | 14/17632 | 0.000761 | 0.008677 | 0.007516 | 4 |
| GO:0045309 | protein phosphorylated amino acid binding | 7/556 | 49/17632 | 0.000813 | 0.009092 | 0.007876 | 7 |
| GO:0004435 | phosphatidylinositol phospholipase C activity | 5/556 | 26/17632 | 0.001163 | 0.012768 | 0.01106 | 5 |
| GO:0004713 | protein tyrosine kinase activity | 14/556 | 177/17632 | 0.001484 | 0.016003 | 0.013862 | 14 |
| GO:0004629 | phospholipase C activity | 5/556 | 28/17632 | 0.001649 | 0.017457 | 0.015122 | 5 |
| GO:0035014 | phosphatidylinositol 3-kinase regulator activity | 4/556 | 17/17632 | 0.001678 | 0.017457 | 0.015122 | 4 |
| GO:0030695 | GTPase regulator activity | 20/556 | 309/17632 | 0.001988 | 0.020331 | 0.017611 | 20 |
| GO:0043548 | phosphatidylinositol 3-kinase binding | 5/556 | 31/17632 | 0.002638 | 0.026516 | 0.022969 | 5 |
| GO:0004620 | phospholipase activity | 9/556 | 99/17632 | 0.004042 | 0.039944 | 0.034601 | 9 |
| GO:0005035 | death receptor activity | 3/556 | 11/17632 | 0.004259 | 0.040086 | 0.034724 | 3 |
| GO:0016004 | phospholipase activator activity | 3/556 | 11/17632 | 0.004259 | 0.040086 | 0.034724 | 3 |
| GO:0016175 | superoxide-generating NADPH oxidase activity | 3/556 | 11/17632 | 0.004259 | 0.040086 | 0.034724 | 3 |

**Supplementary Table 2** KEGG pathway analysis of *LINC02195* in TCGA database (HNSCC).

| ID | Description | GeneRatio | BgRatio | pvalue | p.adjust | qvalue | Count |
| --- | --- | --- | --- | --- | --- | --- | --- |
| hsa05330 | Allograft rejection | 28/331 | 38/7471 | 1.30E-30 | 2.75E-28 | 2.17E-28 | 28 |
| hsa04612 | Antigen processing and presentation | 37/331 | 77/7471 | 3.00E-30 | 3.18E-28 | 2.51E-28 | 37 |
| hsa05332 | Graft-versus-host disease | 26/331 | 41/7471 | 8.49E-26 | 6.00E-24 | 4.73E-24 | 26 |
| hsa04940 | Type I diabetes mellitus | 26/331 | 43/7471 | 5.20E-25 | 2.76E-23 | 2.18E-23 | 26 |
| hsa04514 | Cell adhesion molecules (CAMs) | 42/331 | 144/7471 | 8.11E-24 | 3.44E-22 | 2.71E-22 | 42 |
| hsa05150 | Staphylococcus aureus infection | 28/331 | 56/7471 | 1.02E-23 | 3.60E-22 | 2.84E-22 | 28 |
| hsa05320 | Autoimmune thyroid disease | 27/331 | 53/7471 | 3.43E-23 | 1.04E-21 | 8.20E-22 | 27 |
| hsa04640 | Hematopoietic cell lineage | 34/331 | 97/7471 | 2.40E-22 | 6.35E-21 | 5.01E-21 | 34 |
| hsa04060 | Cytokine-cytokine receptor interaction | 56/331 | 294/7471 | 1.57E-21 | 3.70E-20 | 2.92E-20 | 56 |
| hsa04672 | Intestinal immune network for IgA production | 24/331 | 49/7471 | 3.33E-20 | 7.05E-19 | 5.57E-19 | 24 |
| hsa04658 | Th1 and Th2 cell differentiation | 31/331 | 92/7471 | 7.13E-20 | 1.37E-18 | 1.08E-18 | 31 |
| hsa05416 | Viral myocarditis | 25/331 | 59/7471 | 4.57E-19 | 8.08E-18 | 6.38E-18 | 25 |
| hsa04659 | Th17 cell differentiation | 32/331 | 107/7471 | 1.04E-18 | 1.70E-17 | 1.34E-17 | 32 |
| hsa05140 | Leishmaniasis | 27/331 | 74/7471 | 1.78E-18 | 2.69E-17 | 2.12E-17 | 27 |
| hsa04650 | Natural killer cell mediated cytotoxicity | 33/331 | 131/7471 | 9.25E-17 | 1.31E-15 | 1.03E-15 | 33 |
| hsa05321 | Inflammatory bowel disease (IBD) | 24/331 | 65/7471 | 1.09E-16 | 1.45E-15 | 1.14E-15 | 24 |
| hsa05340 | Primary immunodeficiency | 18/331 | 37/7471 | 2.25E-15 | 2.81E-14 | 2.22E-14 | 18 |
| hsa05168 | Herpes simplex infection | 37/331 | 185/7471 | 3.76E-15 | 4.43E-14 | 3.50E-14 | 37 |
| hsa05323 | Rheumatoid arthritis | 26/331 | 90/7471 | 5.38E-15 | 6.00E-14 | 4.74E-14 | 26 |
| hsa04062 | Chemokine signaling pathway | 37/331 | 190/7471 | 9.22E-15 | 9.77E-14 | 7.71E-14 | 37 |
| hsa04145 | Phagosome | 33/331 | 152/7471 | 1.04E-14 | 1.05E-13 | 8.32E-14 | 33 |
| hsa05310 | Asthma | 16/331 | 31/7471 | 2.53E-14 | 2.44E-13 | 1.93E-13 | 16 |
| hsa04380 | Osteoclast differentiation | 28/331 | 128/7471 | 9.91E-13 | 9.14E-12 | 7.21E-12 | 28 |
| hsa05152 | Tuberculosis | 33/331 | 179/7471 | 1.43E-12 | 1.26E-11 | 9.94E-12 | 33 |
| hsa05169 | Epstein-Barr virus infection | 35/331 | 201/7471 | 1.64E-12 | 1.39E-11 | 1.10E-11 | 35 |
| hsa05322 | Systemic lupus erythematosus | 28/331 | 133/7471 | 2.70E-12 | 2.20E-11 | 1.74E-11 | 28 |
| hsa05166 | Human T-cell leukemia virus 1 infection | 35/331 | 219/7471 | 2.10E-11 | 1.65E-10 | 1.30E-10 | 35 |
| hsa05145 | Toxoplasmosis | 24/331 | 113/7471 | 8.58E-11 | 6.49E-10 | 5.13E-10 | 24 |
| hsa05164 | Influenza A | 29/331 | 171/7471 | 2.86E-10 | 2.09E-09 | 1.65E-09 | 29 |
| hsa04660 | T cell receptor signaling pathway | 22/331 | 101/7471 | 3.21E-10 | 2.27E-09 | 1.79E-09 | 22 |
| hsa05162 | Measles | 24/331 | 132/7471 | 2.48E-09 | 1.69E-08 | 1.34E-08 | 24 |
| hsa04630 | JAK-STAT signaling pathway | 23/331 | 162/7471 | 6.15E-07 | 4.08E-06 | 3.22E-06 | 23 |
| hsa05170 | Human immunodeficiency virus 1 infection | 26/331 | 212/7471 | 2.01E-06 | 1.29E-05 | 1.02E-05 | 26 |
| hsa04620 | Toll-like receptor signaling pathway | 16/331 | 104/7471 | 1.16E-05 | 7.26E-05 | 5.73E-05 | 16 |
| hsa04064 | NF-kappa B signaling pathway | 15/331 | 95/7471 | 1.59E-05 | 9.61E-05 | 7.59E-05 | 15 |
| hsa05133 | Pertussis | 13/331 | 76/7471 | 2.44E-05 | 0.000144 | 0.000113 | 13 |
| hsa05144 | Malaria | 10/331 | 49/7471 | 4.46E-05 | 0.000256 | 0.000202 | 10 |
| hsa05143 | African trypanosomiasis | 8/331 | 35/7471 | 0.000112 | 0.000627 | 0.000495 | 8 |
| hsa05142 | Chagas disease (American trypanosomiasis) | 14/331 | 102/7471 | 0.000147 | 0.000792 | 0.000625 | 14 |
| hsa04621 | NOD-like receptor signaling pathway | 19/331 | 168/7471 | 0.000149 | 0.000792 | 0.000625 | 19 |
| hsa04610 | Complement and coagulation cascades | 12/331 | 79/7471 | 0.000165 | 0.000852 | 0.000673 | 12 |
| hsa05167 | Kaposi sarcoma-associated herpesvirus infection | 20/331 | 186/7471 | 0.000201 | 0.001015 | 0.000801 | 20 |
| hsa05163 | Human cytomegalovirus infection | 22/331 | 225/7471 | 0.000383 | 0.001888 | 0.00149 | 22 |
| hsa04666 | Fc gamma R-mediated phagocytosis | 12/331 | 91/7471 | 0.00063 | 0.003037 | 0.002398 | 12 |
| hsa04662 | B cell receptor signaling pathway | 10/331 | 71/7471 | 0.001055 | 0.004972 | 0.003925 | 10 |
| hsa04625 | C-type lectin receptor signaling pathway | 12/331 | 104/7471 | 0.002064 | 0.00951 | 0.007508 | 12 |
| hsa04611 | Platelet activation | 13/331 | 123/7471 | 0.00301 | 0.013578 | 0.010719 | 13 |
| hsa04623 | Cytosolic DNA-sensing pathway | 8/331 | 63/7471 | 0.006291 | 0.027785 | 0.021936 | 8 |
| hsa05221 | Acute myeloid leukemia | 8/331 | 66/7471 | 0.008321 | 0.036001 | 0.028422 | 8 |
| hsa04670 | Leukocyte transendothelial migration | 11/331 | 112/7471 | 0.010525 | 0.044624 | 0.03523 | 11 |
